# Trimethylguanosine synthase 1 is a novel regulator of pancreatic beta-cell mass and function

**DOI:** 10.1016/j.jbc.2022.101592

**Published:** 2022-01-15

**Authors:** Manuel Blandino-Rosano, Pau Romaguera Llacer, Ashley Lin, Janardan K. Reddy, Ernesto Bernal-Mizrachi

**Affiliations:** 1 Division of Endocrinology, Diabetes and Metabolism, Department of Internal Medicine, Miller School of Medicine, University of Miami, Miami, Florida, USA; 2 Miami VA Health Care System, Miami, Florida, USA; 3 Department of Pathology, Feinberg School of Medicine, Northwestern University, Chicago, Illinois, USA

**Keywords:** diabetes, beta-cell, ER stress, apoptosis, cell cycle arrest, insulin secretion, BrdU, bromodeoxyuridine, CBs, Cajal Bodies, ER, endoplasmic reticulum, GO, gene ontology, HFD, high-fat fed, IR, insulin receptor, RNPs, ribonucleoproteins, snoRNAs, small nucleolar RNAs, snoRNPs, small nucleolar RNPs, snRNAs, small nuclear RNAs, snRNPs, small nuclear RNPs, T2D, Type 2 diabetes, TGS1, Trimethylguanosine synthase 1, TMG, 2,2,7-trimethylguanosine, TMX, tamoxifen, UPR, unfolded protein response

## Abstract

Type 2 diabetes is a metabolic disorder associated with abnormal glucose homeostasis and is characterized by intrinsic defects in β-cell function and mass. Trimethylguanosine synthase 1 (TGS1) is an evolutionarily conserved enzyme that methylates small nuclear and nucleolar RNAs and that is involved in pre-mRNA splicing, transcription, and ribosome production. However, the role of TGS1 in β-cells and glucose homeostasis had not been explored. Here, we show that TGS1 is upregulated by insulin and upregulated in islets of Langerhans from mice exposed to a high-fat diet and in human β-cells from type 2 diabetes donors. Using mice with conditional (*βTGS1KO*) and inducible (*MIP-Cre*^*ERT*^*-TGS1KO*) TGS1 deletion, we determined that TGS1 regulates β-cell mass and function. Using unbiased approaches, we identified a link between TGS1 and endoplasmic reticulum stress and cell cycle arrest, as well as and how TGS1 regulates β-cell apoptosis. We also found that deletion of TGS1 results in an increase in the unfolded protein response by increasing XBP-1, ATF-4, and the phosphorylation of eIF2α, in addition to promoting several changes in cell cycle inhibitors and activators such as p27 and Cyclin D2. This study establishes TGS1 as a key player regulating β-cell mass and function. We propose that these observations can be used as a stepping-stone for the design of novel strategies focused on TGS1 as a therapeutic target for the treatment of diabetes.

Type 2 diabetes (T2D) is a disease of epidemic proportions and a major public health problem with an estimated total cost of $673 billion (1). Type 2 diabetes is characterized by insulin resistance in peripheral tissues such as skeletal muscle and liver and intrinsic defects in β-cell function and mass. β-cells mainly adapt to insulin resistance by increasing function and proliferation, although cell size and apoptosis also play a role (2). However, a decompensated phase is observed when β-cells are unable to respond to the insulin demands required to control glucose homeostasis, resulting in impaired function and deterioration of β-cells mass. Despite several mechanisms that have been proposed, chronic exposure of β-cells to hyperglycemia is supposed to alter the protein folding capacity of the endoplasmic reticulum (ER), resulting in accumulation and aggregation of unfolded proteins that cause ER stress (3).

Trimethylguanosine synthase 1 (TGS1) is an evolutionarily conserved enzyme that mediates the methylation of small nuclear and nucleolar RNAs (snRNAs and snoRNAs), selenoprotein, and telomerase mRNAs and is involved in pre-mRNA splicing, transcription, and ribosome biogenesis (4, 5). Initially, snRNAs and snoRNAs are capped with 7-monomethylguanosine and methylated by TGS1 to form 2,2,7-trimethylguanosine (TMG). The TMG cap modification is highly conserved throughout eukaryotes and is a critical requirement for RNA transport and regulation of splicing (6). More importantly, previous studies have demonstrated that impaired small nuclear ribonucleoproteins (snRNPs) biogenesis affects glucose metabolism and pancreatic development in mice and humans (7). Although very little is known about TGS1 in vertebrates and virtually nothing about TGS1 in β-cells, previous studies indicate that TGS1 could be involved in controlling glucose homeostasis (7, 8, 9, 10, 11). Mice with a conditional deletion of TGS1 in the liver exhibited alterations in liver-specific metabolic pathways followed by impaired hepatic gluconeogenesis (11). Furthermore, higher TGS1 levels were previously observed in the soleus muscle of rats exposed to high sucrose diet-induced insulin resistance (10). More importantly, TGS1 was elevated in cultured myoblasts stimulated with insulin and TGS1 overexpression in rat skeletal muscle, and cultured myoblasts inhibited insulin-stimulated glucose uptake, further supporting the role of TGS1 in regulating insulin sensitivity (10). These findings pointed toward an important role for TGS1 in regulating hepatic gluconeogenesis, insulin resistance, and glucose homeostasis. On the other hand, the role of TGS1 in pancreatic β-cell function is completely unknown.

Given the important role of snRNAs and snoRNAs in β-cells and in pancreas development (7, 8) and the association between TGS1 upregulation in hyperglycemia and the regulation of hepatic glucose output (10, 11), we decided to study TGS1 in β-cells. Herein, we observed that TGS1 levels are increased in high-fat fed (HFD) mice and humans with T2D. Using mice with conditional TGS1 deletion in β-cells (*βTGS1KO*), the present studies show that TGS1 deficiency results in decreased β-cell function and mass due to increased ER stress, apoptosis, and cell cycle arrest. RNA-Sequencing analysis in control and *βTGS1KO* islets at 1 month of age demonstrated differential expression in several ER stress and cell cycle arrest genes. Together, the current data demonstrate the importance of TGS1 levels in β-cells and suggest that controlling TGS1 levels could be a novel therapeutic target to control glucose levels in T2D.

## Results

### Trimethylguanosine synthase 1 levels are elevated in diabetes models and are induced by glucose and insulin in a paracrine/autocrine manner

**Figure 1:**
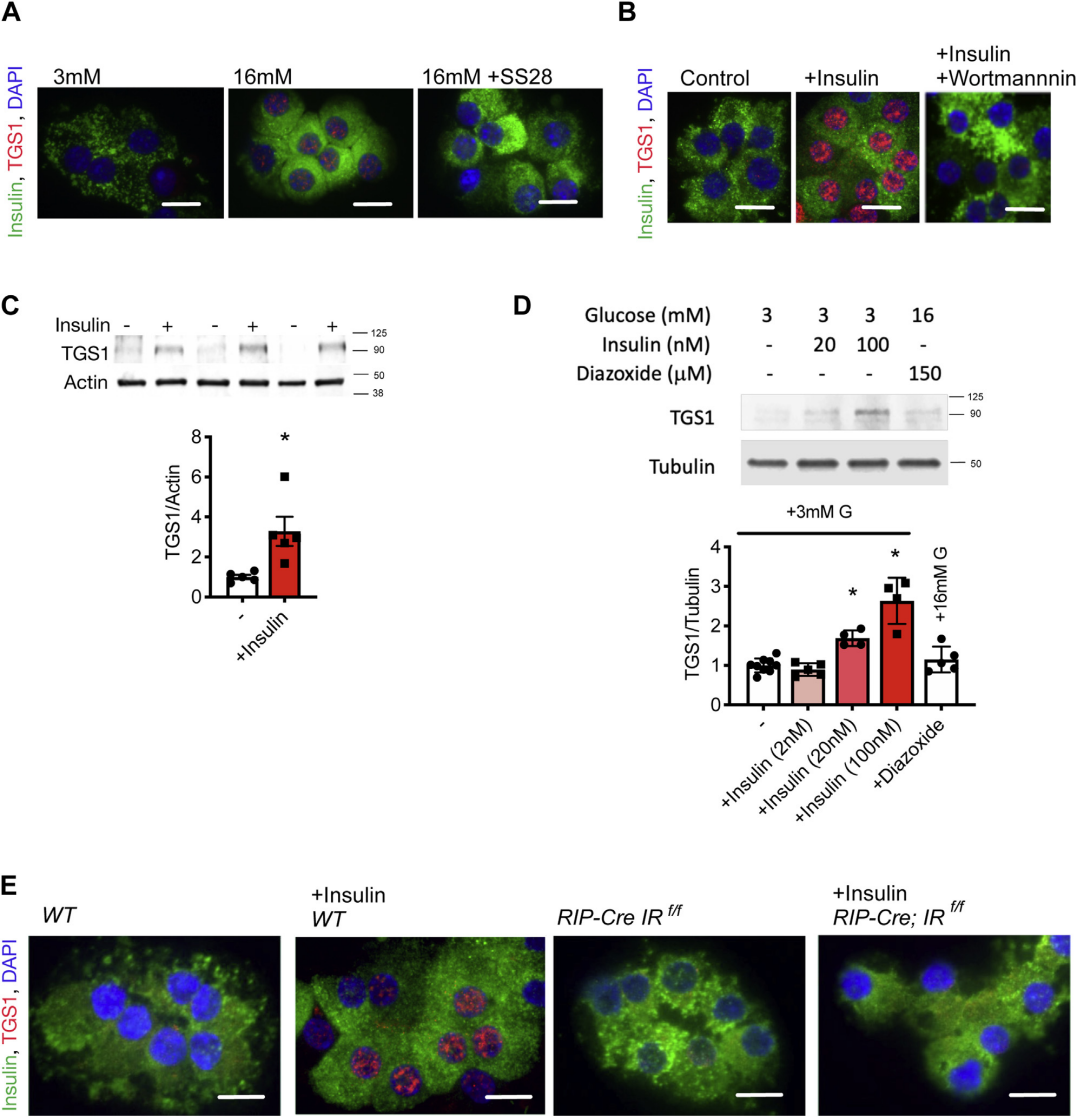
**Insulin regulates TGS1 expression.***A*, immunostaining of insulin (*green*), TGS1 (*red*), and DAPI (*blue*) in dispersed islets from WT mice exposed to 3, 16 mM, and 16 mM glucose +SS28 (1 μM) for 24 h. The scale bar represents 10 μm (n = 3). *B*, immunostaining of insulin (*green*), TGS1 (*red*), and DAPI (*blue*) of dispersed β-cells from WT islets treated with or without insulin (100 nM) and wortmannin (10 nM) for 24 h. The scale bar represents 10 μm (n = 3). *C*, immunoblotting and quantification of TGS1 and actin in WT islets treated with or without insulin (100 nM) at 3 mM glucose for 24 h (n = 4). *D*, immunoblotting and quantification of TGS1 and tubulin in WT islets treated without or with 2, 20, and 100 nM insulin at 3 mM glucose and diazoxide (150 μM) for 24 h (n = 4). *E*, immunostaining for insulin (*green*), TGS1 (*red*), and DAPI (*blue*) in dispersed islets from WT and *RIP-Cre IR*^*f/f*^ mice exposed to insulin (100 nM) for 24 h. The scale bar represents 10 μm. Staining shows a representative image from three independent experiments (n = 3). The data are expressed as means ± SD, ∗*p* < 0.05. TGS1, Trimethylguanosine synthase 1.

To study the regulation of TGS1 by glucose in β-cells, isolated WT islets were treated with 3 or 16 mM glucose for 24 h. Staining of dispersed islets demonstrates that high glucose exposure increases protein levels of TGS1 in β-cells (Fig. 1*A*). To assess the paracrine role of insulin on TSG1 upregulation by glucose, we used Somatostatin-28, a potent inhibitor of insulin secretion. Somatostatin-28 treatment prevented the TSG1 upregulation induced by high glucose, suggesting that high glucose induced TGS1 expression by insulin in a paracrine/autocrine manner (Fig. 1*A*). To test the direct effect of insulin, we treated WT islets with low glucose (3 mM) and insulin (100 nM) for 24 h (Fig. 1*B*). Trimethylguanosine synthase 1 staining and immunoblotting show that insulin treatment induced TGS1 levels in islets from WT mice (Fig. 1, *B* and *C*). To further assess the hypothesis that secreted insulin induces TGS1 in a paracrine fashion, we performed studies with the hyperpolarizing agent diazoxide. The increase in TGS1 by high glucose was blocked by the treatment with diazoxide (Fig. 1*D*), validating the results obtained by somatostatin (Fig. 1*A*). Treatment with the PI3K inhibitor wortmannin also inhibited the induction of TGS1 by insulin (Fig. 1*B*). To further investigate whether the action of insulin was specific to the insulin receptor (IR), two additional experiments were performed. First, TGS1 levels were assessed using different concentrations of insulin (2, 20, and 100 nM). Insulin at 20 nM and 100 nM but not 2 nM were sufficient to significantly increase TGS1 levels (Fig. 1*D* and data not shown for 2 nM). In the second experiment, isolated islets from mice with deletion of the IR in β-cells (*RIP-Cre IR*^*f/f*^) and control littermates were treated with insulin (100 nM) for 24 h. Elevation of TGS1 levels after insulin treatment was observed only in control mice (Fig. 1*E*). These data together indicate that insulin, but not glucose, regulates TGS1 expression.

### Trimethylguanosine synthase 1 is increased in insulin resistance conditions

**Figure 2:**
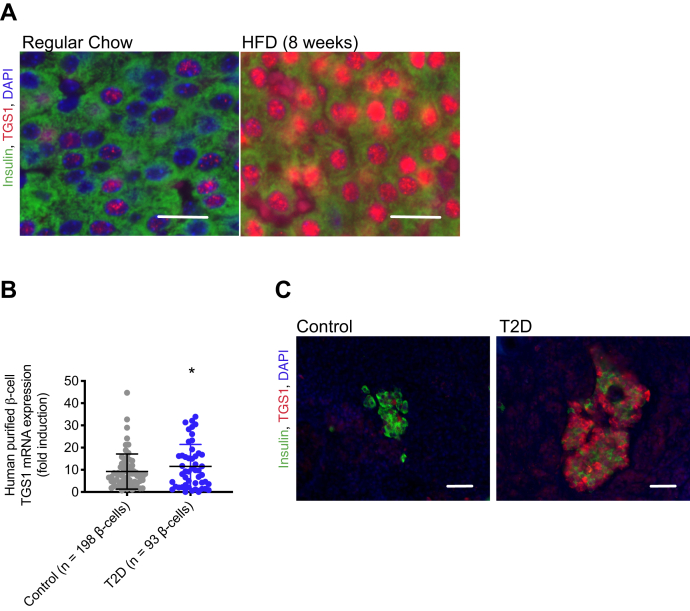
**TGS1 is increased in insulin resistance conditions.***A*, immunostaining for insulin (*green*), TGS1 (*red*), and DAPI (*blue*) in pancreas sections from mice on regular chow or HFD. The Scale bar represents 20 μm. *B*, RNA-Sequencing dataset from healthy (n = 198) and T2D (n = 93), purified human β-cells obtained from public repositories (E-MTAB-5061/5060) (12). *C*, immunostaining for insulin (*green*), TGS1 (*red*), and DAPI (*blue*) in pancreas sections from control and T2D donors. The scale bar represents 100 μm. Staining shows a representative image from three independent experiments. The data are expressed as means ± SD, ∗*p* < 0.05. HFD, high-fat diet; T2D, type 2 diabetes; TGS1, Trimethylguanosine synthase 1.

Consistent with the previous data, TGS1 levels in β-cells are elevated in conditions of HFD-induced insulin resistance and hyperglycemia (mice fed HFD for 8 weeks) (Fig. 2*A*). Assessment of TGS1 expression in purified β-cells (reanalysis of a previously published RNA-Seq dataset (E-MTAB-5061/5060) (12)) and stained pancreatic sections from human donors showed that TGS1 mRNA and protein were increased in T2D donors compared to controls (Fig. 2, *B* and *C*). Moreover, the extent to which this increase in TGS1 plays either a beneficial compensatory role or induces β-cell failure was unexplored.

### Generation of mice with TGS1 inactivation in β-cells

**Figure 3:**
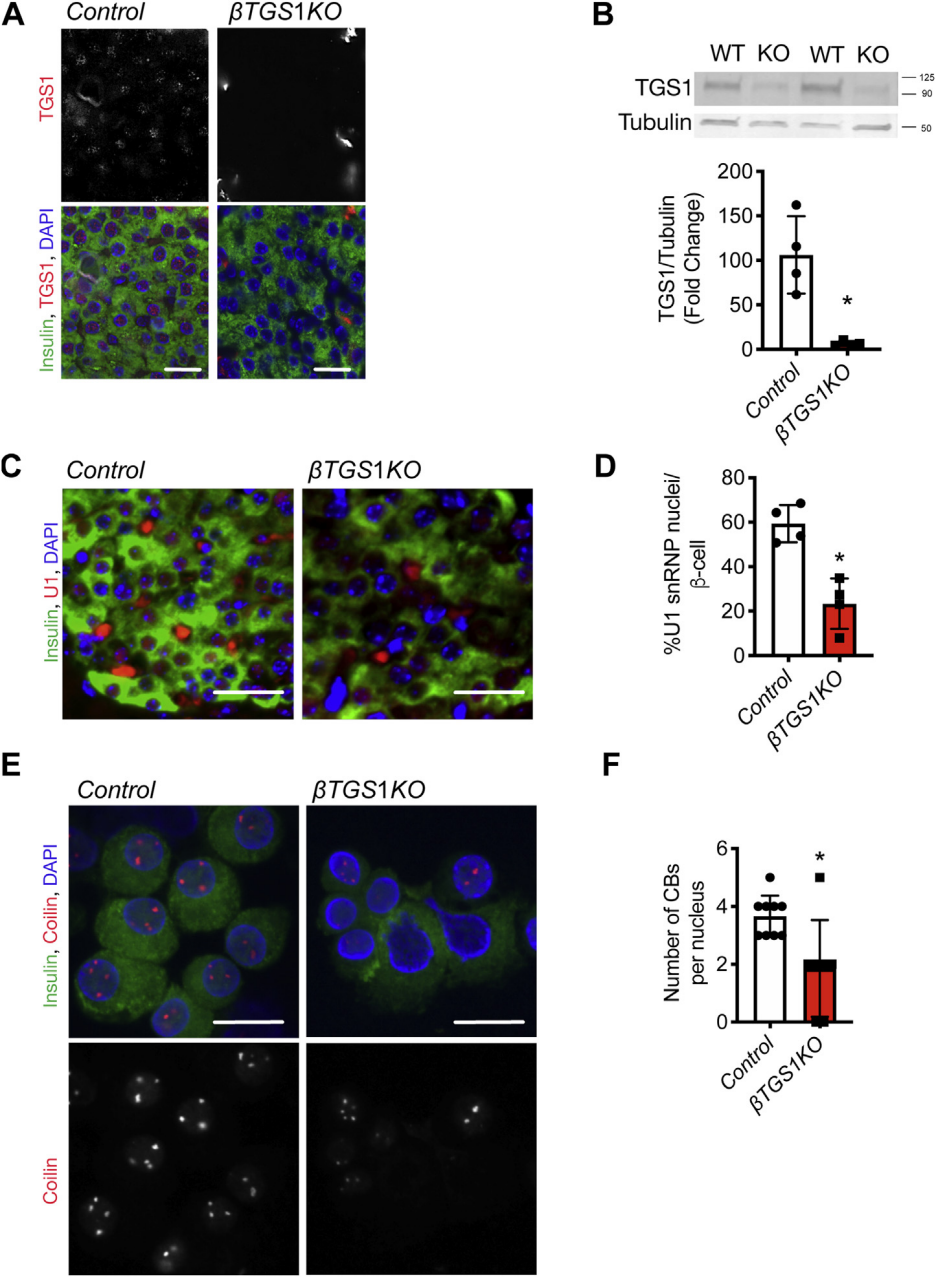
**TGS1 deletion decreased nuclear levels of U1 and coilin and reduced Cajal Bodies number.***A*, immunostaining for TGS1 (*red*), insulin (*green*), and DAPI (*blue*) in pancreas sections from *control* and *βTGS1KO*. The scale bar represents 20 μm. *B*, immunoblotting and quantification of TGS1 and tubulin in isolated islets from control and *βTGS1KO*. Staining (*C*) and quantification (*D*) of nuclear U1 (*red*), insulin (*green*), and DAPI (blue) in pancreas sections from *control* and *βTGS1KO* mice. The scale bar represents 30 μm. *E*, staining for coilin (*red*, *upper* and *white*, *lower*) in dispersed islets from control and *βTGS1KO* mice. The scale bar represents 10 μm. *F*, number of CBs per nucleus in *βTGS1KO* and controls (n = 4). Staining shows a representative image from four independent experiments. The results are expressed as means ± SD ∗*p* < 0.05. CB, Cajol Bodies; TGS1, Trimethylguanosine synthase 1.

To inactivate TGS1 function, we generated mice with homozygous deletion of TGS1 in β-cells by crossing *TGS1*^*f/f*^ (13) with *RIP-Cre* mouse (14) (*βTGS1KO*). Figure 3*A* shows that TGS1 is absent in β-cells from *βTGS1KO* islets, and this was confirmed by immunoblotting (Fig. 3*B*). The TMG cap is synthesized by TGS1 and is abundantly present on snRNAs essential for pre-mRNA splicing (4, 5, 15, 16, 17, 18, 19, 20). Previous studies have shown that the TMG cap provides a signal for the efficient nuclear import of the newly assembled snRNPs (4, 15, 21, 22). To validate the inactivation of TGS1 in β-cells, we assessed U1 snRNP nuclear levels. Immunostaining of pancreas sections from control and *βTGS1KO* mice showed a 30% reduction of nuclear U1 snRNP levels with no differences in total U1 snRNP levels in β-cells from *βTGS1KO* mice (Fig. 3, *C* and *D*). Altered snRNPs translocation to the nucleus has been associated with disruption in Cajal Bodies (CBs) formation (23, 24). To examine CB formation, we assessed coilin levels, a well-known marker of CBs. Coilin levels were decreased, and this was associated with reduced numbers of CBs (Fig. 3, *E* and *F*). Taken together, these experiments validate the inactivation of TGS1 function in *βTGS1KO* mice.

### Constitutive deletion of TGS1 in β-cells results in hyperglycemia

**Figure 4:**
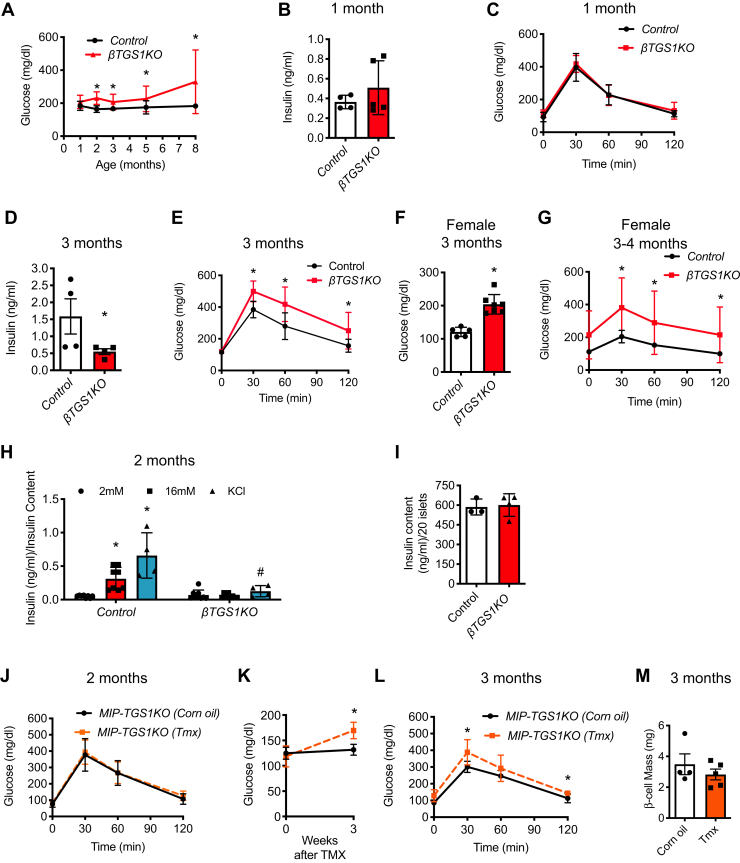
**Constitutive deletion of TGS1 in β-cells results in hyperglycemia.***A*, random fed glucose levels during the first 8 months of age. *B*, random fed serum insulin levels at 1 month of age. *C*, intraperitoneal glucose tolerance test (IPGTT) at 1 month of age. *D*, random fed serum insulin levels at 3 months of age. *E*, intraperitoneal glucose tolerance test at 3 months of age. *F*, random fed glucose levels in females at 3 months of age. *G*, intraperitoneal glucose tolerance test in females at 3 to 4 months of age. *H*, glucose-stimulated insulin secretion (GSIS) *in vitro* using isolated islets from 2-month-old (30 mM KCl). *I*, insulin content from islets used in (*H*) (n = 4). *J*, intraperitoneal glucose tolerance test before TMX injection at 2 months of age. *K*, random fed glucose levels before and 3 weeks post-TMX injection. *L*, intraperitoneal glucose tolerance test 4 weeks (3 months of age) post-TMX injection in *MIP-TGS1KO*. *M*, assessment of β-cell mass 4 weeks after TMX injection. The results are expressed as means ± SD, ∗*p* < 0.05 compared to control, ^#^*p* < 0.05 compared to KCl control. TGS1, Trimethylguanosine synthase 1; TMX, tamoxifen.

*βTGS1KO* mice exhibit a normal response to glucose and normal fed glucose and insulin levels up to 1 month of age (Fig. 4, *A*–*C*). Hypoinsulinemia and hyperglycemia in the fed state were observed in *βTGS1KO* mice after 1 month of age (Fig. 4, *A* and *D*–*G*). At 3 months, male *βTGS1KO* mice exhibit an increase in fed glucose (Fig. 4*A*,) and decrease in insulin (Fig. 4*D*) and impaired glucose tolerance after glucose challenge (Fig. 4*E*). This phenotype was also observed in females, suggesting that sexual dimorphism is not playing a role in the TGS1 deficient mice (Fig. 4, *F* and *G*). Moreover, *in vitro* glucose-stimulated insulin secretion was blunted in islets from *βTGS1KO* mice at 1 month of age and this is not explained by alterations in insulin content (Fig. 4, *H* and *I*). To determine whether the defect in glucose-stimulated insulin secretion was distal to the influx of extracellular calcium, insulin secretion was measured after treatment with KCl. KCl-induced insulin release was reduced in β-cells from *βTGS1KO*, indicating that the defect is at a step distal to depolarization and calcium influx (Fig. 4*H*). To validate the importance of TGS1 in insulin secretion, we deleted TGS1 in two-month-old mice using a mouse model with tamoxifen (TMX) inducible deletion of TGS1 in β-cells (*MIP-Cre*^*ERT*^*-TGS1KO*). These studies show that *MIP-Cre*^*ERT*^*-TGS1KO* mice display elevation in fed glucose at 3 weeks post-TMX injection (Fig. 4, *J* and *K*). In addition, glucose tolerance was impaired in *MIP-Cre*^*ERT*^*-TGS1KO* mice, and this was accompanied by comparable β-cell mass (Fig. 4, *L* and *M*).

### βTGS1KO exhibits a reduction in β-cell mass

**Figure 5:**
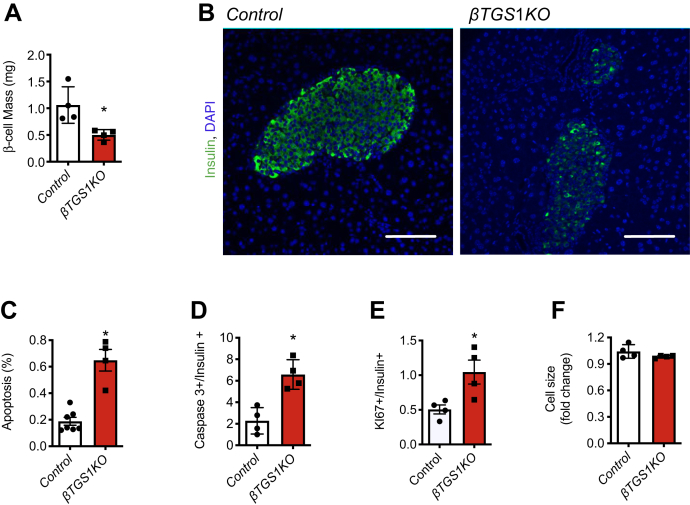
***βTGS1KO* mouse exhibits a reduction in β-cell mass due to increase in apoptosis at 3 months of age.***A*, assessments of β-cell mass and (*B*) representative pictures of islets from *βTGS1KO* and control mice. The scale bar represents 100 μm. *C*, apoptosis in β-cells by TUNEL, (*D*) Caspase 3 positive in insulin-positive cells by FACS, (*E*) proliferation by Ki67 staining, and (*F*) cell size. Values are expressed as means ± SD, ∗*p* < 0.05. FACS, fluorescence-activated cell sorting.

To determine if the defect in insulin secretion results from the alterations in stimulus-secretion coupling and/or a reduction in β-cell mass, we performed assessment of β-cell morphometry. β-cell mass in *βTGS1KO* mice was reduced at 3 months of age (Fig. 5, *A* and *B*). Decrease in β-cell mass in *βTGS1KO* mice was associated with increase in β-cell apoptosis assessed by TUNEL and active Caspase 3 measured by fluorescence-activated cell sorting (Fig. 5, *C* and *D*). This was accompanied by increase in β-cell proliferation assessed by Ki67 staining (Fig. 5*E*). No changes in β-cell size were observed in *βTGS1KO* mice (Fig. 5*F*).

### RNA-Sequencing studies in βTGS1KO reveal alterations in markers of ER stress

**Figure 6:**
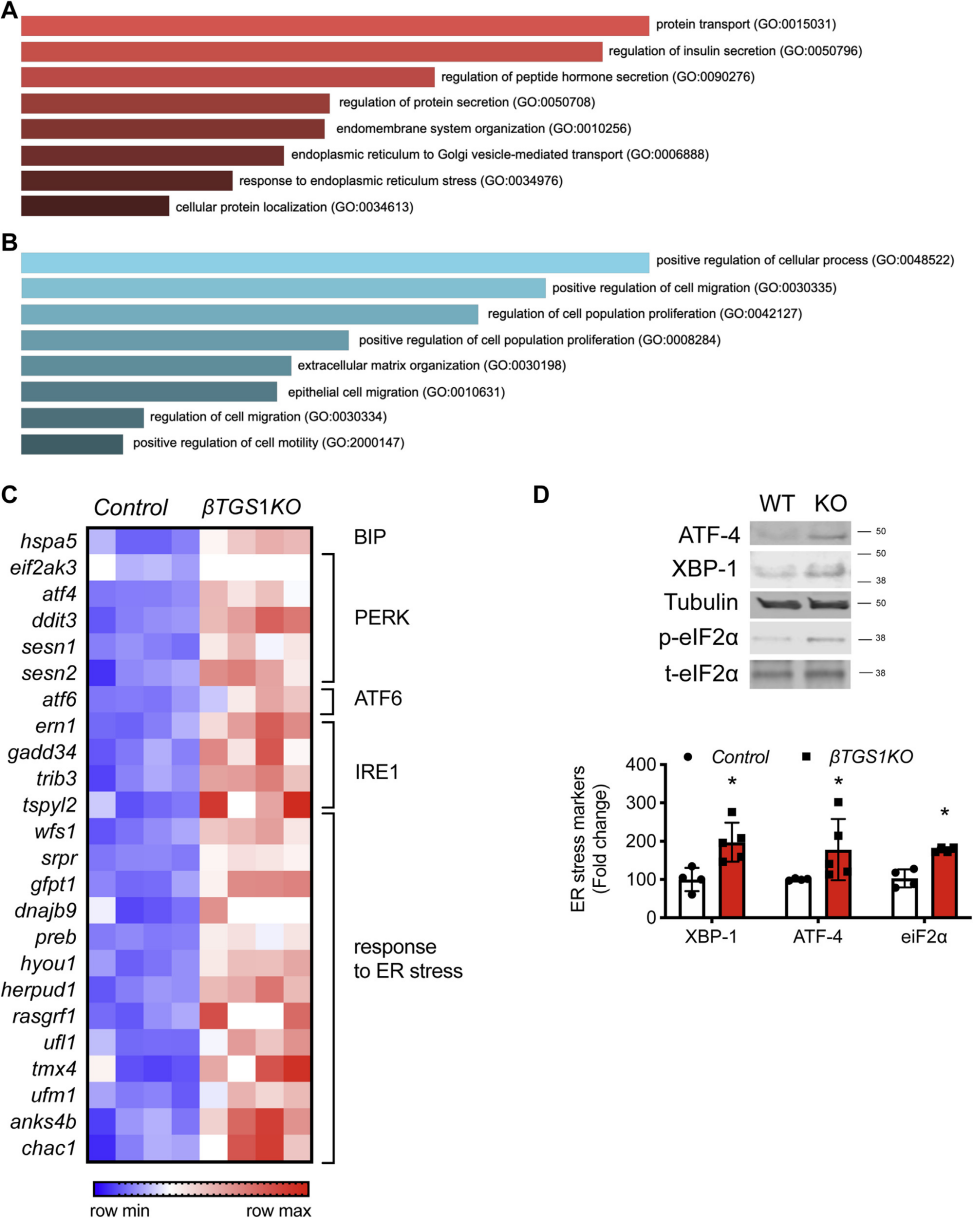
***β-cells with deletion of TGS1* exhibit signs of ER stress.** Gene ontology-driven pathway analysis of differentially expressed genes with (*A*) >1.5-fold change and (*B*) <0.5-fold change between *βTGS1KO* and control islets at 1 month of age. *C*, gene-expression heatmap of the differentially expressed genes related to unfolded protein response (UPR) and the response to ER stress in *βTGS1KO* islets compared to control. Genes are represented in rows and mice in columns. *D*, immunoblotting and quantification of ATF-4, XBP-1 respect to tubulin, and phospho respect to total eiF2α in islets from *control* and *βTGS1KO* mice at 1 month of age. The data are expressed as means ± SD ∗*p* < 0.05. ER, endoplasmic reticulum; TGS1, Trimethylguanosine synthase 1.

**Figure 7:**
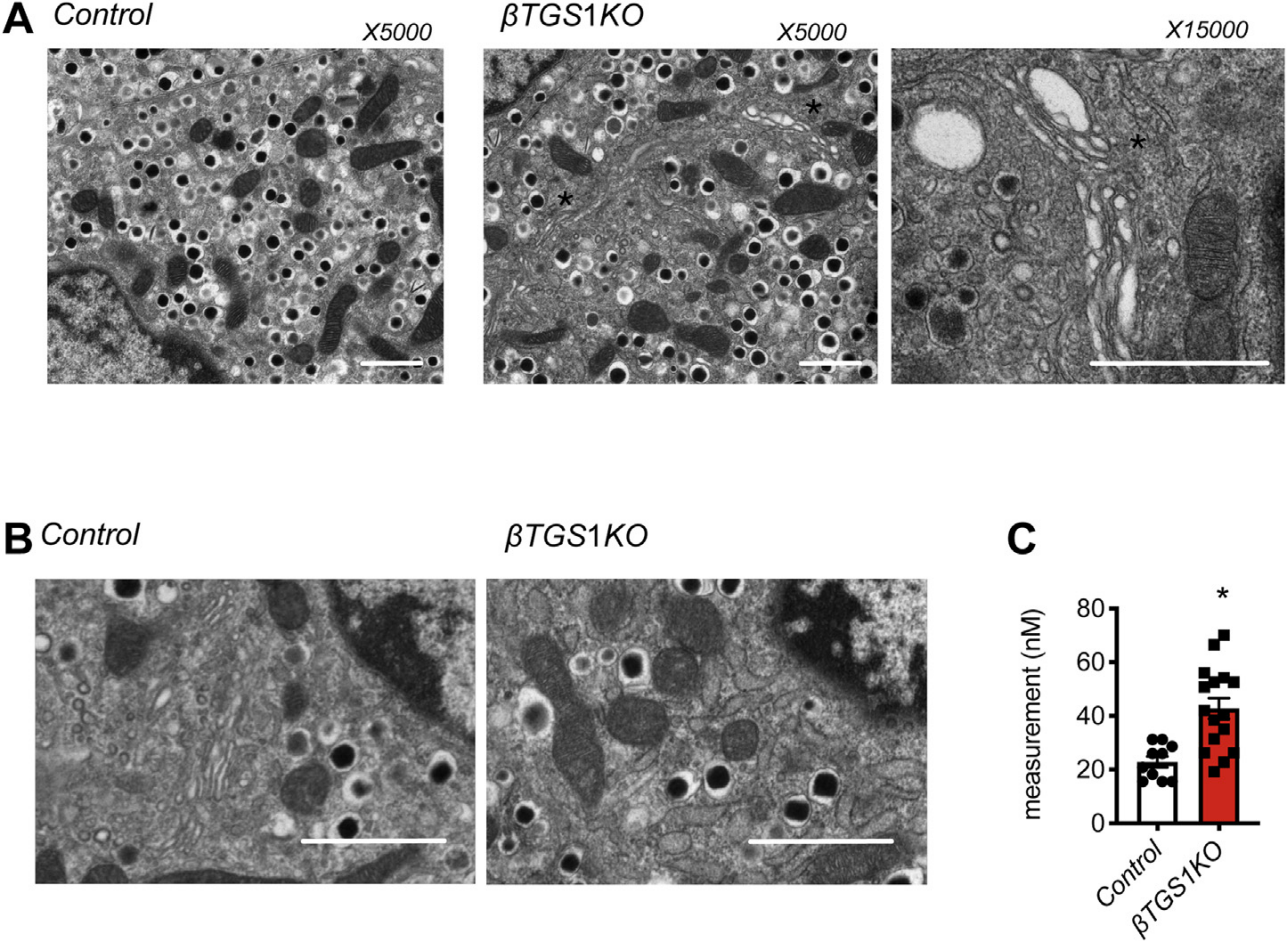
***β-cells with deletion of TGS1* show enlarged ER.***A*, electron microscopy images showing enlarged ER in β-cells from *βTGS1KO* mice at 1 month of age. Magnification: ×5000 and ×15,000. The scale bar represents 1 μm. The images are representative of islets from three animals. *Asterisks* indicate areas showing ER enlarged. *B*, representative electron microscopy images showing dilation of the ER in β-cells from *βTGS1KO* mice compared to controls. *C*, quantitative analysis representing the extent of ER dilation. The scale bar represents 1 μm. The data are expressed as means ± SD ∗*p* < 0.05. ER, endoplasmic reticulum; TGS1, Trimethylguanosine synthase 1.

To test how TGS1 regulates β-cell apoptosis, we performed RNA-Seq in islets from *βTGS1KO* before the development of hyperglycemia and reduction of β-cell mass (1 month-old). We used EnrichR (25) to perform gene ontology (GO)-term analysis on the full list of genes differentially expressed. Gene ontology-term analysis revealed increased expression patterns (>1.5-fold change) in genes involved in protein transport, insulin, protein hormone and protein secretion, and ER stress (Fig. 6*A*). Decreased expression (<0.5-fold change) of genes associated with cellular processes including cell migration, proliferation, migration, and extracellular matrix organization (Fig. 6*B*). Unbiased pathway enrichment analysis indicated that genes related to unfolded protein response (UPR) were increased in *βTGS1KO* (z-score = 5.61). The mRNA of BIP (hspa5), a protein that interacts with the three UPR activator proteins, PERK, ATF6, and IRE1α acting as a repressor of the UPR and playing a role in the apoptosis process was significantly increased in *βTGS1KO* islets (Fig. 6*C*). It was not unexpected to see that genes related with the three separate branches of the ER response were also increased: 1. PERK (eif2ak3), atf4, ddit3 (CHOP/GADD153), and Sestrins); 2. ATF6; and 3. IRE1α (ern1, gadd34, trib3, and tspyl2). In addition, other top differentially expressed genes related to ER response, such as wfs1, dnajb9, or hyou1 are also observed in Figure 6*C*. Given the importance of ER stress in β-cells, we validated the abnormalities in UPR pathway gene expression by immunoblotting. Figure 6*D* shows a 2-fold increase in XBP-1, confirming the activation of UPR pathways *via* the induction of PERK. eIF2α phosphorylation and ATF4 were elevated in islets from *βTGS1KO* (Fig. 6*D*). Electron microscopy shows that ER of β-cells from *βTGS1KO* mice was enlarged (Fig. 7, *A* and *B*). Measurements of ER dilation showed a 2-fold increase in β-cells from *βTGS1KO* when compared to controls (Fig. 7, *B* and *C*).

### βTGS1KO exhibits abnormalities in cell cycle progression

**Figure 8:**
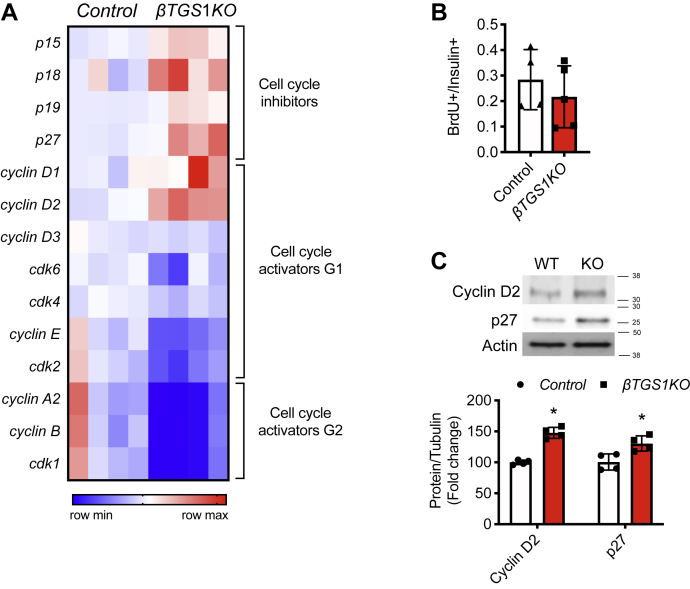
***β-cells with deletion of TGS1* exhibit cell cycle arrest.***A*, gene-expression heatmap of the differentially expressed genes related to cell cycle inhibitors, cell cycle components related to G1 phase, and cell cycle components related to G2 phase from the RNA-Seq of *βTGS1KO* islets compared to control. Genes are represented in *rows* and mice in *columns*. *B*, assessments of proliferation by BrdU staining by FACS. *C*, immunoblotting and quantification for Cyclin D2, p27, and actin (n = 4). The values are expressed as means ± SD, ∗*p* < 0.05. FACS, fluorescence-activated cell sorting; TGS1, Trimethylguanosine synthase 1.

The morphometric analysis suggested that the increase in proliferation was not accompanied by increases in β-cell mass, indicating that cell cycle progression abnormalities could be involved. To assess this, we compared the results of Ki67 (marker of all phases of the cell cycle, Fig. 5*E*) with bromodeoxyuridine (BrdU) (S phase). The fraction of BrdU in β-cells was similar between *βTGS1KO* and control mice (Fig. 8*B*). To assess the mechanisms of cell cycle abnormalities, we analyzed the RNA-Seq data. mRNAs of G1-S cell cycle inhibitors p18, p19, and p27 were increased in *βTGS1KO* islets (Figs. 6*B* and 8*A*). Analysis of mRNAs of cell cycle components that drive G1-S transition showed increase in cyclins D1 and D2, necessary regulators for cell cycle entry but significantly decreased levels of cyclin D partners cdk6 and cdk4 in *βTGS1KO* mice. Other G1-S transition genes, cyclin E and cdk2, were also reduced in *βTGS1KO* mice (Fig. 8*A*). Cyclin A, cyclin B and cdk1 mRNAs, genes implicated in S, G2 and M phase, were also decreased in *βTGS1KO* mice (Fig. 8*A*). The increases in Cyclin D2 and p27 mRNAs, two key regulators of G1-S transition in β-cells, were validated by immunoblotting (Fig. 8*C*). Taken together, these experiments suggest that deletion of TGS1 in β-cells entered the cell cycle but exhibited abnormal transition and completion of the cell cycle.

## Discussion

The present studies assessed the role of TGS1 in β-cells. We demonstrated that TGS1 is upregulated in β-cells in mouse and human models of T2D. The importance of TGS1 was further assessed in mice with constitutive and inducible inactivation of this gene in β-cells. These experiments showed that TGS1 inactivation in mouse β-cells results in hyperglycemia and glucose intolerance as a result of a reduction in β-cells mass. The alterations in glucose homeostasis and insulin secretion were validated in mice with inducible deletion of TGS1 in β-cells (*MIP-Cre*^*ERT*^*-TGS1KO*). In conclusion, the present work identifies TGS1 as an important regulator of β-cells mass and function and demonstrates that this enzyme is important for adaptation to insulin resistance.

The current experiments showed that *βTGS1KO* mice maintain glucose homeostasis during the first month of life and slowly develop hyperglycemia (Fig. 4*A*). We found that reduced β-cell mass played a major role in the alteration in glucose homeostasis in these mice. The defect in β-cell mass was in part explained by induced β-cell apoptosis. A major finding of these experiments was the identification of TGS1 as a regulator of the UPR and ER stress. This conclusion was supported by RNA-seq studies and validated by immunoblotting and electron microscopy. Our data show an increase in mRNA of BIP (hspa5), a major regulator of UPR pathways. This was accompanied by mRNA expression for components of the PERK, ATF6, and IRE1α pathways. Electron microscopy showed dilated ER validating the changes in mRNA and protein. The effect of TGS1 on survival is consistent with the increase in TUNEL observed in TGS1 deficient blastocysts, suggesting that TGS1 might alter transcription of critical survival genes (13). In line with our findings, a main target of ATF-4, ddit3/CHOP mRNA was increased in *βTGS1KO* (Fig. 6*C*).

The morphometric analysis suggests that the increase in proliferation is not sufficient to compensate, indicating a discrepancy between apoptosis and proliferation or alterations in the cell cycle. The increase in Ki67 without changes in BrdU suggest that β-cell from *βTGS1KO* failed to progress to the S phase (Figs. 5*E* and 8*B*). These cell cycle profile abnormalities were further analyzed by RNA-Seq expression of the cell cycle profiles. These studies show an increase in G1-S transition drivers, including Cyclin D1 and 2, and cell cycle inhibitors that regulate G1-S transition including p18, p19, and p27. These changes were accompanied by a decrease in cyclin D complex partners cdk6 and cdk4; other G1-S transition genes, cyclin E and cdk2; and G2 and M phase components, cyclin A, cyclin B, and cdk1 mRNAs (Fig. 8*A*). These experiments suggest that β-cell with TGS1 deletion entered the cell cycle but did not progress through the different phases. An interesting possibility is that genes involved in cell cycle arrest can contribute to the abnormalities of cell cycle in *βTGS1KO* mice. CHOP, a very well-known marker of ER stress, is also a DNA-damage-inducible gene as gadd34. These genes were increased in the RNA-Seq data from *βTGS1KO* islets (Fig. 6*C*) and could suppress cell growth as described (26). Further, mRNA of genes related to growth arrest and DNA-Damage-inducible such as sesn1 and sesn2 (Sestrin 1 and 2) (27, 28, 29), are also upregulated in *βTGS1KO* islets (Fig. 6*C*). This suggests that TGS1 can modulate cell cycle not only by regulating cell cycle machinery but also by upregulating genes involved in cell cycle arrest.

Previous work has uncovered some of the molecules and pathways controlling β-cells in T2D. The present work adds TGS1 as a novel regulator of β-cells from mouse models of hyperglycemia and in human islets from T2D. The mechanisms for the increase in TGS1 levels in models of diabetes are not completely identified, but we show that insulin in a paracrine fashion is involved. Our studies with Somatostatin-28, diazoxide, and wortmannin support the conclusion that insulin but not glucose or cosecreted species is responsible for the increase of TGS1 in β-cells after glucose stimulation (Fig. 1, *A*, *B* and *D*). The studies using lower insulin concentrations and experiments in islets from *RIP-Cre IR*^*f/f*^ suggest that insulin *via* the IR and not the IGF-1 receptor triggers the increase in TGS1 (Fig. 1, *D* and *E*). More experiments will be needed to demonstrate whether low TGS1 levels play a role in the phenotype described in mice with deletion of IR in β-cells (30).

The important role of TGS1 in insulin secretion was also validated in *MIP-Cre*^*ERT*^*-TGS1KO* after TMX injection (Fig. 4, *J*–*L*) and *βTGS1KO* at 1 month of age with no changes in insulin content or β-cell mass (Fig. 4, *I*–*M*). The mechanisms underlying the defect in insulin secretion after TGS1 deletion are unclear, but a blunted response to KCl in *βTGS1KO* islets suggests that the defect was distal to depolarization and calcium influx. Assessment of differentially expressed genes involved in secretory pathways could lead to uncovering how TGS1 regulates insulin secretion. Downregulated genes such as *gata4*, *cldns* (claudins), *onecut1*, and *sphk1* among others could be explored in future experiments. Alternatively, XBP-1 mRNA splicing (31) and IRE1-mediated degradation of insulin mRNA (32) could also contribute to the defect in insulin secretion in *βTGS1KO* mice. Independently of ER stress, TGS1 could be regulating the trafficking and/or fusion of insulin granules with the plasma membrane. Future experiments will be needed to test some of these hypotheses.

Major open questions after these studies are how TGS1 regulates the UPR, cell cycle components, and insulin secretion. Answer to these questions will require a significant effort but these studies provide a stepping-stone to uncover novel mechanisms in β-cells. Trimethylguanosine synthase 1 regulates the methylation of snRNA and snoRNA, and this mediates the biogenesis of snRNPs and small nucleolar RNPs (snoRNPs) complexes by binding to the survival of motor neurons complex (snRNPs) and NOP proteins (snoRNPs) in the cytoplasm and nucleus, respectively. Small nuclear RNPs and snoRNPs are major constituents of the spliceosome and contribute to restoring homeostasis to RNA metabolism upon recovery from the stress. Future experiments can be designed to examine the role of mRNA splicing, transcription, and ribosome production in the observed β-cell phenotypes. Altered snRNPs biogenesis has been associated with defects in CBs biogenesis leading to splicing defects (24). Small nucleolar RNAs also alter mitochondrial metabolism, modulate glucose-stimulated insulin secretion and responses to oxidative stress in islets, thereby providing a link between TGS1 and insulin secretion (33). Future studies could be designed to assess how these mechanisms regulate insulin secretion, ER stress, and β-cell proliferation.

Here, we identify TGS1 as a novel regulator of β-cell mass and function. Our results suggest that an increase in TGS1 levels could play a beneficial role in the adaptation of β-cell to insulin resistance and diabetes. In addition, these studies show that TGS1 regulates ER stress and cell cycle progression controlling β-cell mass. Together, these data support the concept that elevation of TGS1 levels in β-cell and insulin-sensitive tissues could be a common marker of hyperglycemia/insulin resistance in diabetes. These studies strongly demonstrate the importance of TGS1 levels in β-cells and suggest that controlling TGS1 levels could be a novel therapeutic target to control glucose levels in T2D.

## Experimental procedures

### Animals and treatments

RIP-Cre (14) and mice with targeted deletion of TGS1 (9, 10, 11, 13) have been previously described. Studies were performed on mice on C57BL6J background. For these experiments, *RIP-Cre TGS1*^*+/+*^, *TGS1*^*f/f*^, and *TGS1*^*f/+*^ were used as controls. Given the limitations of the Cre models used in our studies, including potential recombination in the hypothalamus (*RIP-Cre*), we used *MIP-Cre*^*ERT*^ mice line (34), and *MIP-Cre*^*ERT*^*-TGS1KO* mice injected with corn oil and *TGS1*^*f/f*^ injected with TMX were used as controls. Because there are some concerns with the GH minigene in the *MIP-Cre*^*ERT*^, we validated key findings in *TGS1*^*f/f*^ mice crossed to *INS-Cre* knock-in (KI) mice (35) (data not shown). To delete IR in β-cell (*RIP-Cre IR*^*f/f*^), we generated mice with homozygous deletion of IR in β-cells by crossing *IR*^*f/f*^ (36) with *RIP-Cre* mouse (14). Results of the experiments are shown for male mice, but phenotypes were validated in female mice. Ages are shown in figure legends. All animals were maintained on a 12 h light–dark cycle. All the procedures were approved by University of Miami IACUC committee and performed in accordance with University of Miami Animal Care Policies and the GUIDE for the care and use of laboratory animals.

### Metabolic studies

Blood glucose levels were determined from blood obtained from the tail vein using Contour glucometer (Bayer). Glucose tolerance tests were performed on animals fasted overnight by intraperitoneally injecting glucose (2 mg/kg). Plasma insulin concentrations were determined using a Mouse ultrasensitive Insulin ELISA kit (ALPCO).

### Islets studies

After islet isolation (37), islets were maintained at 37 °C in an atmosphere containing 20% oxygen and 5% CO_2_. Insulin, wortmannin, diazoxide, KCl, and TMX were purchased from Sigma. Insulin secretion from isolated islets was assessed by static incubation (37). Briefly, after overnight culture in RPMI containing 5 mM glucose and 10% fetal bovine serum, islets were precultured for 1 h in Krebs–Ringer medium containing 2 mM glucose. Groups of 20 islets in quadruplicates were then incubated in Krebs–Ringer medium containing 2 or 16 mM glucose for 40 min. Secreted insulin in the supernatant and insulin content was then measured using Mouse Ultrasensitive Insulin ELISA kit (ALPCO Immunoassays) and normalized to insulin total content.

### Immunoblotting

Islets from an individual mouse (150–300 islets) were lysed in lysis buffer (125 mM Tris, pH 7, 2% SDS, and 1 mM DTT) containing a protease inhibitor cocktail (Roche Diagnostics). Protein quantity was measured by a bicinchoninic acid assay method, and 20 to 40 μg of protein were loaded on SDS–PAGE gels and separated by electrophoresis. Separated proteins were transferred onto PVDF membranes (Millipore) overnight. After blocking for 1 h in Intercept Blocking Buffer from LI-COR , the membranes were incubated overnight at 4 °C with a primary antibody diluted in the same buffer followed by 1 h incubation at room temperature with secondary antibodies from the same company. Antibodies used for immunoblotting are included in Table S1, and membranes were developed using LI-COR Odyssey FC. Band densitometry was determined by measuring pixel intensity using NIH Image J software/Fiji (v2.1.0/1.53c (ref. (38)) freely available at http://rsb.info.nih.gov/ij/index.html) and normalized to tubulin, actin, or total protein in the same membrane. Images have been cropped for presentation. Full-size images for the most important western blots are available from the authors on request.

### Fluorescence-activated cell sorting

After overnight culture in RPMI containing 5 mM glucose, islets were dispersed into a single-cell suspension and fixed with BD Pharmingen Transcription Factor Phospho Buffer Set (BD Biosciences). The dispersed cells were incubated overnight with conjugated antibodies at 4 °C. Dead cells were excluded by Ghost Dye Red 780 (Tonbo), and signal intensity from single stained cells and GFP was analyzed by mean fluorescent intensity in insulin-positive cells using BD LSR II (BD Biosciences). Antibodies used are included in Table S1.

### RNA-Sequencing library preparation, sequencing, and data analysis

This data has been deposited in the GEO repository (pending). RNA Quality Control and DNase Treatment: A total of eight mice islets RNA samples were submitted to Ocean Ridge Biosciences for mRNA-Sequencing. Total RNA was quantified by absorbance measurement and assessed for quality on a 1% agarose – 2% formaldehyde RNA Quality Control gel. The RNA was then digested with RNase free DNase I (Epicenter; Part # D9905 K) and repurified using Agencourt RNAClean XP beads (Beckman Coulter; Part # A63987). The newly digested RNA samples were then quantified by absorbance measurement. The newly digested RNA samples were then quantified by absorbance measurement and checked for quality.

Library Preparation: Amplified cDNA libraries suitable for sequencing were prepared from 250 ng of DNA-free total RNA using the TruSeq Stranded mRNA Library Prep (Illumina Inc; Part # 20020595). The quality and size distribution of the amplified libraries were determined by chip-based capillary electrophoresis (Bioanalyzer 2100, Agilent Technologies). The libraries were quantified using the KAPA Library Quantification Kit (Kapa Biosystems).

Sequencing: The eight libraries were pooled at equimolar concentrations and sequenced in a total of 3 runs on the Illumina NextSeq 500 sequencer using two Mid Output v2 150 cycle kits (part# FC-404–2001) and one High Output v2.5150 cycle kit (part# 20024907). In each case, the libraries were sequenced with 76 nucleotide paired-end reads plus 8 nucleotide dual-index reads on the instrument running NextSeq Control Software version 2.2.0.4. Real-time image analysis and base calling were performed on the instrument using the Real-Time Analysis software version 2.4.11. Generation of FASTQ files: Base calls from the NextSeq 500 Real-Time Analysis were converted to sequencing reads in FASTQ format using Illumina’s bcl2fastq program v2.17.1.14 with default settings. Sequencing adapters were not trimmed in this step.

Gene ontology analysis was performed using EnrichR (25). Gene ontology Function process output comma-delimited (CSV) files were saved, and all those pathways having an adjusted *p*-value <0.05 were selected in the analysis. We selected commonly and highly ranked GO pathways, highly enriched in genes derived from the differential gene expression analysis.

### Immunofluorescence and morphometry

Formalin-fixed pancreatic tissues were embedded in paraffin and sectioned. Immunofluorescence staining was performed using primary antibodies described on Table S1. Fluorescent images were acquired using a microscope (Leica DM5500B) with a motorized stage using a camera (Leica Microsystems, DFC360FX), interfaced with the OASIS-blue PCI controller and controlled by the Leica Application Suite X (LAS X). β-cell ratio assessment was calculated by measuring insulin and acinar areas using Adobe Photoshop in five insulin-stained sections (5 μm) that were 200 μm apart. To calculate β-cell mass, β-cell to acinar ratio was then multiplied by the pancreas weight. Assessment of proliferation was performed in insulin- and Ki67-stained sections and included at least 3000 cells per animal. Apoptosis was determined using TUNEL assay (ApopTag Red *in Situ* Apoptosis Detection Kit, Chemicon) in insulin-stained sections. At least, 3000 β-cells were counted for each animal. Cell size was determined by immunostaining sections measuring the areas of individual β-cells from different experimental groups using NIH Image J software/Fiji. For dispersed cell staining, the islets were gently dispersed after 5 min incubation with trypsin–EDTA (0.25% trypsin and 1 mM EDTA) in Hanks’ balanced salt solution without Ca^2+^ and Mg^2+^ (Gibco Invitrogen) at 37 °C followed by fixation in 4% methanol-free formaldehyde onto poly-l-lysine-coated slides. All the morphologic measurements were performed in blinded manner.

### Electron microscopy

Ultrastructural characterization by transmission electron microscopy was performed after overnight culture (in RPMI containing 5 mM glucose at 37 °C). Islets were then fixed with 2% glutaraldehyde and then dehydrated and embedded in Epon by the Transmission Electron Microscopy Core at University of Miami. Ultrathin sections were stained with uranyl acetate and lead citrate, and images were recorded digitally using a Philips CM-100 electron microscope.

Images were reviewed to assess dilation of the ER. The length was drawn longitudinally, and the center of the length was used as the reference point to measure the width of the ER, indicative of the relative amount of dilation. We randomly picked 4 to 6 ER within each cell with 3 to 4 cells reviewed in each group. Measurements are reported as μm.

### Human organ donors

Human pancreas tissue samples (from the head of the pancreas, non-diabetic individuals and T2 diabetic individuals (n = 4), male and female, ages = 15–52 years-old) from the Human Islet Cell Processing Facility at the Diabetes Research Institute, University of Miami.

### Statistical analysis

Data are expressed as mean ± SD. Student’s unpaired *t* test was used to assess statistical difference between 2 groups using Prism version 9 (GraphPad Software). Comparison between more than 2 groups was performed using 2-way ANOVA with repeated measures followed by post hoc 2-tailed Student’s *t* tests. The results were considered statistically significant when the *p* value was equal than 0.05.

## Data availability

All relevant data are available from the authors on request.

## Supporting information

This article contains supporting information.

## Conflict of interest

The authors declare that they have no conflicts of interest with the contents of this article
