# Supplementary material for: Trimethylguanosine synthase 1 is a novel regulator of pancreatic beta-cell mass and function
Source: J Biol Chem. 2022 Jan 15;298(3):101592. doi: 10.1016/j.jbc.2022.101592 (PMC8861161; doi:10.1016/j.jbc.2022.101592)
Supplement: Supplemental Table S1 [file mmc1.docx]

**Supplemental Table 1. Antibodies**
